# Permanent Tooth Agenesis and Associated Dental Anomalies among Orthodontically Treated Children

**DOI:** 10.3390/children10030596

**Published:** 2023-03-21

**Authors:** Shirley Schonberger, Rana Kadry, Yehoshua Shapira, Tamar Finkelstein

**Affiliations:** The Maurice and Gabriela Goldschleger School of Dental Medicine, Department of Orthodontics, Tel Aviv University, Tel Aviv 69978, Israel

**Keywords:** infra-occluded teeth, impacted teeth, orthodontic treatment, peg-shaped incisors, tooth agenesis

## Abstract

(1) Background: Tooth agenesis is one of the most common developmental dental anomalies often affecting the maxillary incisors area and premolar regions. (2) Purpose: The aim of this study was to assess the prevalence and distribution of permanent tooth agenesis and the associated dental abnormalities among orthodontically treated children. (3) Materials and Methods: This study was carried out utilizing 3000 pretreatment records of children who underwent orthodontic treatment, 1780 (59%) females and 1220 (41%) males, aged 10–25 years (mean age 15 years). Tooth agenesis and other dental anomalies were surveyed using their panoramic radiographs, according to gender, pattern, and location. The level of statistical significance was set at *p* < 0.05 using *t*-test or Chi-Square tests. (4) Results: The total number of missing teeth, excluding third molars, was 518 (17%) found in 326 (11%) children. The majority were the maxillary lateral incisors, which was 176 teeth (34%) (*p* < 0.05). Of them, 111 (63%) were in females, and 65 (37%) were in males. The second most common missing tooth was mandibular second premolars, which was 137 teeth (26%), followed by missing 73 (14%) maxillary second premolars. Impacted teeth had the highest associated dental anomaly prevalence (14.3%), while transposition showed the lowest anomaly prevalence (0.5%). (5) Conclusions: A prevalence of 11% for tooth agenesis was detected in this study. More teeth were missing in the maxilla compare with the mandible. A significant association was found between missing maxillary lateral incisors and missing premolars (*p* < 0.05). Associated dental anomalies included an increased number of peg-shaped maxillary lateral incisors, palatally displaced and impacted maxillary canines, ectopic teeth, and infra-occluded (submerged) primary second molars.

## 1. Introduction

Permanent tooth agenesis is a very common developmental dental anomaly in children most often detected in the maxillary anterior segment and in the mandibular and maxillary premolar regions [1]. Tooth agenesis is a result of disturbances during the initial stages of tooth formation and the initiation and proliferation stages [2]. This can be due to genetic or environmental causes, resulting in abnormal tooth development. The main cause for tooth agenesis is heredity affected by an autosomal dominant gene with incomplete penetrance [3,4]. Tooth development is affected by genes, and agenesis is a result of mutations in MSX1, PAX9, AXIN2, TGFA, and some other genes [5]. It has also been suggested that tooth agenesis could be an expression of an evolutionary process (phylogenetic tendency), where jaws and the number of teeth are being reduced [6]. As a general rule, a missing tooth is always the most distal tooth of each group of teeth [7]. Therefore, the most common missing tooth in the molar group is the third molar, in the premolar group, it is the second premolar, and in the incisor group, it is the lateral incisor [8]. In contrast, teeth that are rarely missing include the central incisors, canines, and first molars. It has been reported that when a primary tooth is missing, its permanent successor will also be absent [8].

Agenesis can be described by several terms. The most frequently used term is hypodontia, where a patient presents with one to five teeth missing in the dental arch, excluding third molars. When six or more permanent teeth are congenitally missing the term is oligodontia. While anodontia refers to an extremely rare condition in which there is a complete absence of teeth in the dental arches [1]. The glossary of prosthodontic terms (GPT-9) uses the same terminology for missing teeth with slightly different definitions. The definitions are classified according to the number of teeth being missing and by the size of teeth. The first term, hypodontia, is defined as the congenital absence of one or more, but not all, of the normal complement of teeth. The second term, oligodontia, is defined as the formation of less than a full complement of teeth, with many such teeth that are smaller than normal. The third term, anodontia, is defined as a rare dental condition characterized by the congenital absence of all teeth, both deciduous and permanent [9]. Other terms in the literature describing the reduction in the number of teeth are congenitally missing teeth, aplasia of teeth, absence of teeth, agenesis, or lack of teeth. Anodontia and oligodontia are frequently associated with unusual systemic abnormalities, such as congenital syndromes, ectodermal dysplasia, and cleft lip and palate [10].

The hypodontia prevalence of permanent teeth varies according to ethnic origin, different populations, and different regions in the world. It ranges between 2.3% and 11.3% in Caucasian populations [11], reported to be 2.6% in Arab orthodontic children in Israel [12], 4.6% in Turkish orthodontic patients [13], 8.5% in Japanese orthodontic children [14], and as high as 14.7% in Hungarian orthodontic patients [15].

In the deciduous dentition, hypodontia is a much less common condition than in the permanent dentition. Similar to the permanent dentition, the hypodontia prevalence of deciduous teeth varies according to ethnic origin. It was reported that in Caucasian population the prevalence was about 1%, and a higher prevalence was reported in Japanese population [16]. The most common missing teeth in the deciduous dentition are the maxillary lateral incisors and mandibular central incisors. Usually, the agenesis appears in a unilateral pattern and with no significant difference regarding gender [17].

Tooth agenesis is often associated in a pattern with other dental anomalies in the same individual. The association between agenesis of teeth to other dental anomalies was investigated by Baccetti [18]. He found a significant association between missing second premolars and other dental anomalies, such as the small size of maxillary lateral incisors, the submergence of primary molars, enamel hypoplasia, and palatally displaced maxillary canines. These findings suggested a common genetic origin for agenesis of teeth and all these other dental anomalies. By contrast, no association was found between supernumerary teeth to aplasia of second premolars or to any other anomaly, which suggests that the anomaly of supernumerary teeth is a separate entity of different origin [18].

An association was found between the agenesis of teeth to several other dental anomalies. These include taurodontism, reduced tooth length, ectopic eruption, reduction in tooth size, changes in tooth morphology, and enamel hypoplasia. Schalk-van der Weide et al. found in their study that taurodontism and a reduced tooth length occur in higher prevalence in patients with oligodontia. The explanation that was suggested for these findings was that an ectodermal defect and a manifestation of developmental instability might cause taurodontism and the reduced root length in patients with oligodontia [19]. Another common dental anomaly associated with hypodontia is the ectopic position of the permanent teeth. This association might be the result of lack of guidance of the ectopic teeth due to the absence of the adjacent teeth that are usually available to guide the ectopic teeth during eruption. Tooth agenesis is also associated with several clinical features, such as microdontia and transposition of permanent teeth [17].

Although the association was reported in several studies, little information is available on the distribution and pattern of tooth agenesis associated with other dental abnormalities. The major purpose of this present study was to prove the association between tooth agenesis and other dental anomalies and to describe several clinical management options. Hence, this study was undertaken to determine the distribution and pattern of tooth agenesis and the association between tooth agenesis with other dental anomalies among orthodontically treated children.

## 2. Materials and Methods

Clinical examination, facial, and intraoral photographs, diagnostic panoramic radiographs, and dental models of 3000 consecutively treated children from a university orthodontic clinic were utilized. It consisted of 1780 (59%) females and 1220 (41%) males in the age range of 10–25 years (mean 15 years), all of Caucasian Israeli ethnical origin. These records were collected for diagnostic purposes, routinely taken prior to orthodontic treatment between 1996 and 2019, unrelated to the present investigation. The data were recorded according to gender, age, number of missing teeth, and their location. Two examiners evaluated each panoramic radiograph separately in order to avoid misdiagnosis of missing teeth or dental anomalies. Inclusion criteria were complete records with high-quality photographs and diagnostic radiographs. Exclusion criteria were incomplete records, a history of traumatic dental avulsion, previous orthodontic or surgical treatment in either dental arches, extraction of permanent teeth, and congenital craniofacial disorders or syndromes.

All panoramic radiographs were taken in the radiologic department of the dental school, using the same equipment and radiation. In addition to missing teeth, seven associated anomalies were identified in the radiographs. The first anomaly was supernumerary teeth, and it was defined as the presence of more teeth than the usual number. The second anomaly was a peg-shaped maxillary lateral incisor, which was defined as a small cone-shaped tooth with incisal mesio-distal width smaller than its cervical width with a narrowing in diameter from the cervix to the incisal edge. The third anomaly was ectopic teeth that were defined as teeth erupting in an abnormal position. The fourth anomaly was impactions. An impacted tooth was defined as a tooth that fails to erupt into the mouth. The fifth anomaly was submergence or infraocclusion. A submerged or infra-occluded tooth was defined as a primary tooth that fails to reach the occlusal surface of the adjacent teeth. The sixth anomaly was a retained tooth, which was defined as failure of a primary tooth to exfoliate at the appropriate time. The last anomaly was the transposition of teeth, which was defined as an interchange in position of two adjacent permanent teeth in the same quadrant of the dental arch. The diagnostic criteria and the prevalence of the associated anomalies are presented in Table 1 and Table 2, respectively.

Prior to the inclusion in the study, an informed consent was obtained from the parent/guardian of every child. This study was approved by the Institutional Ethics Review Committee of Tel Aviv University.

The data were analyzed using the SPSS software package (Statistical Package for Social Sciences, Version 20.0, SPSS Inc., Chicago, IL, USA). Statistical significance was set at *p* < 0.05 using *t*-test or Chi-Square tests.

## 3. Results

**Figure 1:**
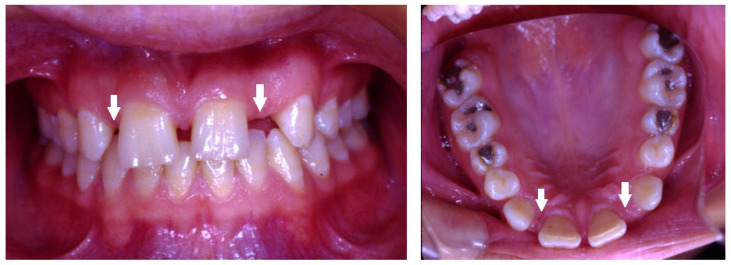
Bilateral missing maxillary lateral incisors in a 12-year-old girl (arrows).

**Figure 2:**
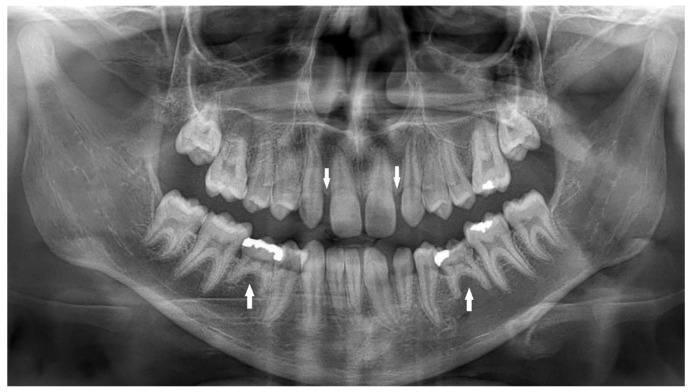
Bilateral missing maxillary lateral incisors and mandibular second premolars in a 13-year-old girl (arrows).

**Figure 3:**
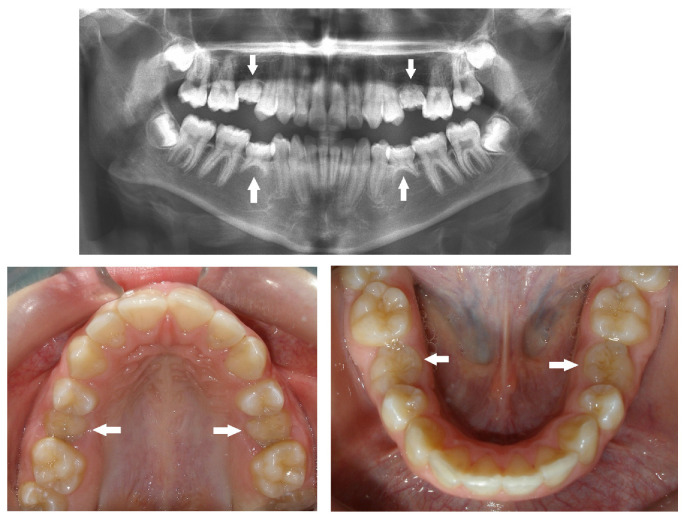
A 13-year-old girl with bilateral missing maxillary and mandibular second premolars (arrows). Bilateral infra-occluded (submerged) maxillary and mandibular primary second molars (arrows).

A total of 518 (17%) missing teeth, excluding third molars, were detected in 326 (11%) children, 187 (57%) in females and 139 (43%) in males. The great majority of missing teeth were the maxillary lateral incisors, which included 176 teeth (34%) (*p* < 0.05), as presented in a 12-year-old girl who was too shy to smile openly (Figure 1), of them 111 (63%) teeth in females and 65 (37%) in males (*p* < 0.05). The second most common missing teeth were the mandibular second premolars, which included 137 teeth (26%), as presented in a 13-year-old girl (Figure 2). Of them, 71 (52%) were in females, and 66 (48%) were in males. This was followed by 73 (14%) missing maxillary second premolars; of them, 49 (67%) were in females, and 24 (33%) were in males (Figure 3) (Table 3).

**Figure 4:**
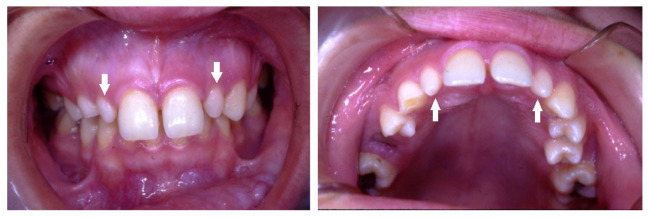
Bilateral peg-shaped maxillary lateral incisors (arrows).

**Figure 5:**
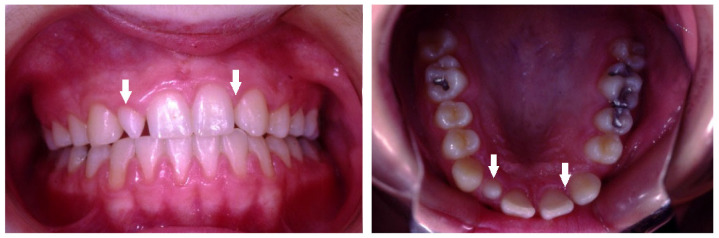
Peg-shaped right lateral incisor and missing left lateral incisor (arrows).

**Figure 6:**
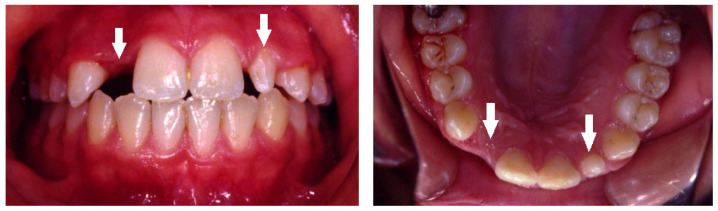
Missing right lateral incisor and peg-shaped left lateral incisor (arrows).

In addition to the missing teeth, we detected an increased prevalence of small and peg-shaped maxillary lateral incisors (Figure 4, Figure 5 and Figure 6), together with a general reduction in tooth size, as well as delayed dental development, especially of the mandibular second premolars and delayed eruption time.

**Figure 7:**
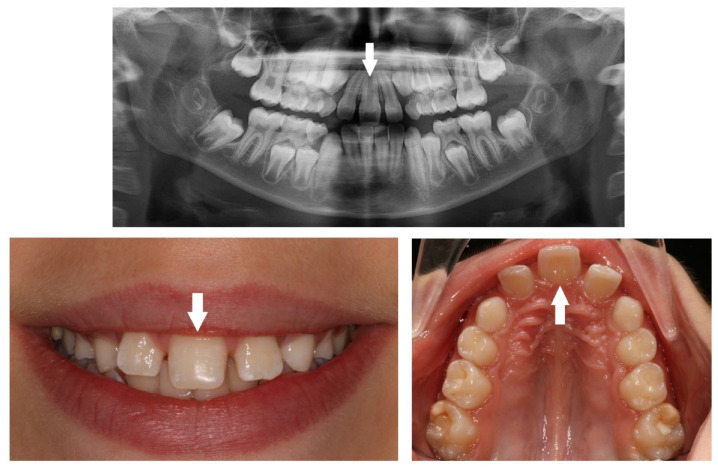
A 10-year-old girl with only one maxillary central incisor in the midline (arrow).

**Figure 8:**
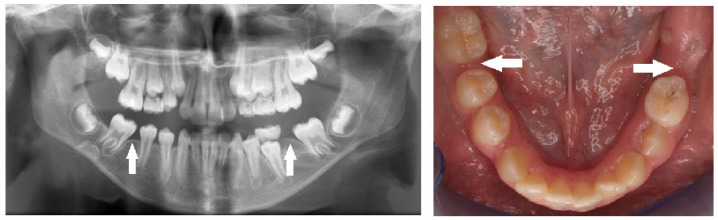
An 11-year-old girl with bilateral missing mandibular permanent first molars (arrows).

Additionally, rare tooth agenesis, including 61 missing first premolars, of which 34 (56%) were from the maxilla and 27 (44%) were from the mandible, was found. In addition, 41 permanent canines were absent, 25 (61%) in the maxilla and 16 (39%) in the mandible. Similarly, 30 (97%) mandibular incisors were found to be missing, with no side preference. Extremely rare cases were detected, including one child presented with only one maxillary incisor in the midline (solitary median maxillary incisor) while the other central incisor was missing (Figure 7). Another child had bilateral missing permanent first molars in the mandibular arch (Figure 8) (Table 4).

**Figure 9:**
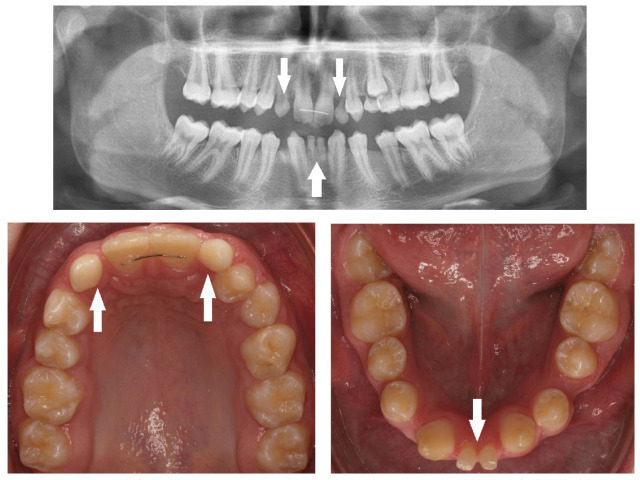
Bilateral missing maxillary lateral incisors and canines, with retained primary canines (arrows). Missing mandibular central and lateral incisors, with retained primary central incisors (arrows).

Associate dental anomalies, which included a combination of bilateral missing maxillary lateral incisors and canines, with retained primary canines, and missing four mandibular incisors, with retained primary central incisors, were detected in a 14-year-old boy (Figure 9).

**Figure 10:**
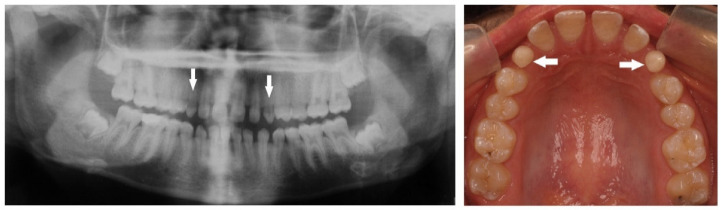
Bilateral missing maxillary canines and retained primary canines (arrows).

Additionally, maxillary bilaterally missing permanent canines with retained primary canines were detected in a 13-year-old girl (Figure 10).

Seven associated dental anomalies were identified in our study and their prevalence is shown in Table 2. Impacted teeth (14.3%) had the highest prevalence anomaly, followed by ectopic (13.8%), and retained teeth (11.5%), while infra-occluded (submerged) teeth (0.7%) and transposition (0.5%) showed the lowest prevalence (Table 2).

**Figure 11:**
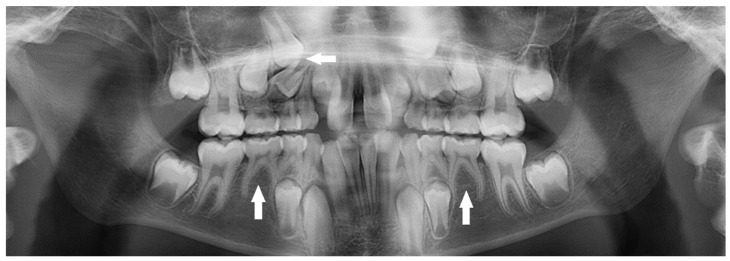
A 10-year-old girl with bilateral missing mandibular second premolars and transposition of maxillary right canine and first premolar (arrows).

**Figure 12:**
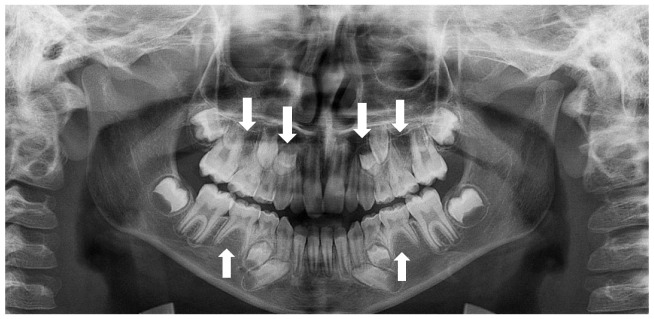
A nine-year-old boy with bilateral missing maxillary and mandibular second premolars and bilateral transposition of maxillary canine and first premolar (arrows).

Peg-shaped maxillary lateral incisors were associated with tooth agenesis, and in the case of unilateral agenesis of maxillary lateral incisor, the contralateral tooth most often was a peg-shaped tooth (Figure 4, Figure 5 and Figure 6). In the posterior region where second premolars were missing a large number of primary second molars were in infraocclusion (submerged) (Figure 3). In some cases, transposition of a maxillary canine and a first premolar was detected together with missing mandibular second premolars (Figure 11). In cases of severe hypodontia, when a bilateral tooth that appears in both jaws was missing, the associated anomaly was also found in a bilateral pattern. For example, in a case that the maxillary and mandibular second premolars were missing bilaterally, an associated anomaly of transposition of maxillary canine and first premolar was found bilaterally as well (Figure 12).

## 4. Discussion

Permanent tooth agenesis is a regular developmental congenital dental anomaly in children, frequently affecting the maxillary anterior region, the “esthetic zone”, and the mandibular and maxillary posterior segments. It is a result of some disturbances in the early stages of dental formation and influenced by genetic and environmental factors [20]. The prevalence of tooth agenesis revealed in this study of orthodontically treated children was 11%, with a higher incidence in females; however, it does not necessarily reflect the prevalence of missing teeth in the general population.

Disagreement exists in the literature regarding the tooth that is most frequently missing. Previous studies on the prevalence of tooth agenesis based their conclusions on ethnic differences in various regions around the world. Reports for Asian ethnicity indicated that the mandibular second premolars and the mandibular incisors are most often the missing teeth [14,21,22]. By contrast, the mandibular second premolars and the maxillary lateral incisors were found to be the most frequent missing teeth for the Caucasian population [23]. For the Druze population living in Jordan, the teeth that were most commonly missing were the maxillary lateral incisors and canines. It was suggested that the different ethnicity of the Druze due to their consanguineous marriages, which led to their genetic isolation, expressed an absence of different teeth than in other populations. [24].

The teeth that were found to be the most commonly absent in our study were the maxillary lateral incisors (54%), followed by the mandibular second premolars (42%), which is in agreement with Kokich [25]. The number of male patients was smaller than female patients, which is related to more female patients seeking orthodontic treatment than male patients.

A higher female prevalence of tooth agenesis was found in the present study, similar to previous reports, indicating more missing teeth in females than in males [11,12]. A much higher prevalence of tooth agenesis (59%) was detected in the maxillary arch compared with the mandibular arch (41%), similar to reports by Alsoleihat [24], Muller [26], Mani [27], and Tunis [28]. This can partially be explained by the different growth and development of the maxilla compared with the mandible [28]. A similar association between dental anomalies in the maxilla and orofacial clefts with agenesis of the lateral incisors at the cleft area was reported [29].

Interestingly, we found a significantly more bilaterally missing maxillary lateral incisors than unilateral agenesis, which was in agreement with previous reports [11,30]. In addition, bilateral agenesis of the mandibular second premolars, as well as the maxillary second premolars, was more common than unilateral agenesis of these teeth. It was reported in a previous study that bilateral agenesis of teeth was significantly more common (80%) than unilateral agenesis (20%) [31]. The different prevalence between unilateral and bilateral agenesis might be explained by different expression of the gene responsible for tooth agenesis [3]. Additionally, in several cases, when the agenesis of second premolars appears in both jaws and bilaterally, the associated anomaly appears in the same bilateral pattern as well (Figure 12). This suggests a common bilateral pattern of agenesis of teeth and the associated dental anomalies in severe hypodontia. It may also suggest a common genetic origin for agenesis of teeth and other developmental dental anomalies. A similar association pattern was found in a study that investigated the patterns of congenitally missing teeth in Japanese orthodontic patients with bilateral missing of mandibular second premolars. In that study, it was reported that significantly increased prevalence rates of bilateral tooth agenesis were observed in the presence of bilateral missing of second mandibular premolars [32].

Tooth agenesis is often associated with a special pattern of other dental anomalies in the same patient. A pattern of association between various dental anomalies were described where individuals with missing third molars presented higher prevalence of missing other permanent teeth [33,34]. Similarly, an association was found between second premolar agenesis and agenesis of other permanent teeth with other dental anomalies [18,35], which is in agreement with our study.

It was found that children with missing maxillary lateral incisors have an increased prevalence of reduction in tooth size, microdontia, and peg-shape lateral incisors unilaterally or bilaterally, similar to our findings. Additionally, delayed dental development is quite common, especially of the mandibular second premolars [36]. Tooth agenesis isolated to the maxilla (missing maxillary lateral incisors) is often associated with small or peg-shaped lateral incisors (Figure 4, Figure 5 and Figure 6). By contrast, tooth agenesis isolated to the mandible (missing mandibular second premolars) is often associated with retained and infraocclusion (submerged) primary molars (Figure 3). Other associated dental anomalies are palatally displaced and impacted permanent canines, submerged primary teeth associated with agenesis of premolars, and supernumerary teeth.

In a recent study by Kadry et al. [37], it was reported that maxillary lateral incisors were the most often absent teeth among the Arab population living in Israel, known for their close relatives marriages (consanguinity). The highest associated dental anomaly found in this group was tooth impaction (13.5%) similar to findings on tooth impaction (14.3%) in our study.

Dental anomalies, such as tooth agenesis, present clinical management challenges to the clinicians. Missing teeth cause spacing issues between the teeth in the dental arch, in particular in the anterior region of the maxillary arch, the “esthetic zone” (Figure 1). It results in severe disturbing esthetics and functional and psychological problems [38]. For most patients, esthetics are crucial to ensure their well-being and self-esteem. Any irregularity of the dentition, especially missing teeth in the maxillary anterior region that affects beauty is undesirable. Children with anterior missing teeth and spaces who are dissatisfied with the position of their front teeth feel ashamed and refrain from smiling, or they cover their mouths while talking or smiling, a condition that influences their well-being and may alter their social lives. Tooth agenesis in the maxillary anterior region also influences the skeletal craniofacial growth of the patient. It was reported that it can reduce the alveolar process development and the soft tissue profile [39]. The diagnosis of tooth agenesis should be performed as early as possible to prevent esthetic and functional problems in the dentition and carry out the most suitable treatment plan.

In the presence of tooth agenesis, there are two major options of treatment. The first option is to close the space and replace the missing tooth with an adjacent tooth. This option, as suggested by Zachrisson et al. [40], Kokich and Kinzer [41], can be applied as canine substitution in congenital missing of the maxillary lateral incisor. The second option is to open or keep the space and replace the missing tooth with a tooth-supported restoration or with an implant-supported restoration [42,43,44]. The selection of the suitable treatment plan for a specific patient depends on several parameters, such as the initial malocclusion of the patient and the condition of the teeth that are adjacent to the missing tooth. Treatment of permanent tooth agenesis, either by closing or opening space for a prosthetic solution, such as a Maryland bridge in the maxillary anterior segment, an implant, or a bridge in the premolar area as previously illustrated [31], is a challenge for all professional specialists. Space closure of the missing lateral incisors by canine substitution was suggested also by Zachrisson et al. [40]. When the treatment of choice is canine substitution, the malocclusion of the patient, the profile, the canine shape and color, and the lip level should be taken into consideration [41].

Alternatively, missing maxillary lateral incisors spaces can be opened for restorative replacement, either by tooth supported restoration or by implant supported restorations, as offered by Kokich et al. [42]. Several options are available for replacing missing maxillary lateral incisor by tooth supported restorations. These options include resin-bonded fixed partial denture, cantilevered fixed partial denture, and conventional full-coverage fixed partial denture [43]. When implant supported restorations are considered, the replacement of missing maxillary lateral incisor can be performed by a single implant. This is much more challenging to the prosthodontist and the orthodontist because it is performed in the esthetic zone and only at the end of growth and development of the patient [44]. Anyway, in each replacement or restorative option, it requires a multidisciplinary approach as it is mainly an esthetic problem in the anterior region of the mouth, “the esthetic zone”, while in the premolar region, it is more of a prosthetic functional problem to replace the missing teeth. Joint efforts of an orthodontist, pediatric dentist, prosthodontist, and an experienced oral surgeon are required when an auto transplantation of a tooth bud (usually a premolar) into the missing tooth space to replace maxillary incisors in growing individuals is considered. It has been reported that tooth bud auto-transplantation can also preserve the alveolar bone in the region of transplantation [45], a preferred procedure compared with an implant, especially in the anterior region of the maxillary arch.

## 5. Conclusions

An overall 11% prevalence for permanent tooth agenesis was detected in orthodontically treated children in this study. A higher incidence was found in the maxillary anterior region with more teeth that were missing in the maxilla compared with the mandible. A higher prevalence of missing teeth was found in females compared to males. The maxillary lateral incisors followed by the mandibular second premolars were found to be the teeth that were most frequently missing. A significant association was found between missing maxillary lateral incisors and missing premolars with a higher prevalence of bilateral missing teeth pattern compared with unilateral missing teeth. A high prevalence of associated dental anomalies was found, mainly impacted teeth (14.3%) that had the highest prevalence anomaly, followed by ectopic (13.8%) and retained teeth (11.5%), while peg-shaped maxillary lateral incisors (1.8%), infra-occluded (submerged) teeth (0.7%), and transposition (0.5%) showed the lowest prevalence. A possible common bilateral pattern of agenesis of teeth and the associated dental anomalies was found in severe hypodontia.

The importance of analyzing dental anomalies together with permanent teeth agenesis is to raise the awareness of the clinician to the problems this phenomenon may create to the practitioner in clinical practice and the importance of diagnosing the problem early in order to prevent future complications. The presence of associations between different tooth anomalies is highly relevant for the clinician. The early diagnosis of tooth agenesis may provide an indication of the increased risk for other associated dental anomalies.

## Figures and Tables

**Table 1:** Diagnostic criteria of dental anomalies in the study.

Dental Anomaly	Diagnostic Criteria
Supernumerary	Presence of extra teeth more than the full complement of teeth.
Missing	Congenital absence of permanent teeth.
Peg-shaped	Small conical tooth with a narrowing in diameter from the cervix to the in-cisal edge.
Impaction	Failure of tooth eruption into the oral cavity.
Transposition	Two adjacent permanent teeth that switched their position within the same quadrant of the dental arch.
Ectopic	Eruption of teeth not in their normal position.
Submergence	A primary tooth positioned under the occlusal surface of the adjacent teeth.
Retained	A primary tooth that fails to exfoliate at the appropriate time.

**Table 2:** Prevalence of associated dental anomalies.

	Peg-Shape Lateral Incisors		Supernumerary Teeth		Ectopic Teeth		Impacted Teeth		Submerged Teeth		Retained Teeth		Transposition	
	No.	%	No.	%	No.	%	No.	%	No.	%	No.	%	No.	%
Total	54	1.8	26	0.9	414	13.8	431	14.3	21	0.7	344	11.5	16	0.5

**Table 3:** Distribution of tooth agenesis by gender.

Gender	Children		Children with Missing Teeth		Missing Maxillary Lateral Incisors		Missing Mandibular Second Premolars		Missing Maxillary Second Premolars	
	No.	%	No.	%	No.	%	No.	%	No.	%
Male	1220	41	139	43	65	37	66	48	24	33
Female	1780	59	187	57	111	63	71	52	49	67
Total	3000	100	326	100	176	100	137	100	73	100

**Table 4:** Distribution of rare tooth agenesis by jaws.

Jaws	Agenesis of First Premolars		Agenesis of Permanent Canines		Agenesis of Central Incisors		Agenesis of First Molars	
	No.	%	No.	%	No.	%	No.	%
Maxilla	34	56	25	61	1	3	0	0
Mandible	27	44	16	39	30	97	2	100
Total	61	100	41	100	31	100	2	100

## Data Availability

Data will be made available upon request.
